# 

**Published:** 1895-06

**Authors:** 


					﻿THE
Southern Medical Record.
A Monthly Journal of Medicine and Surgery.
Vol. XXV.	ATLANTA, GA., JUNE, 1895.	No. 6?
EDITORS:
A. W. GRIGGS, M. D.	. WILLIS F. WESTMORELAND, M. D.
j. McFadden gaston, m. d.	louis h. jones, m. d.
DAN. H. HOWELL, M. D., Business Manager.
Entered at the Post-office at Atlanta, Ga., as Second-Class Matter.
The Foote & Davies Cp., Atlanta, Ga.
				

